# Low-energy versus middle-energy extracorporeal shockwave therapy for the treatment of snapping scapula bursitis

**DOI:** 10.12669/pjms.332.12262

**Published:** 2017

**Authors:** Nihat Acar

**Affiliations:** 1 Nihat Acar, Department of Orthopaedics and Traumatology, Catalca Ilyas Cokay Hospital, Catalca, Istanbul, Turkey

**Keywords:** Extracorporeal shockwave therapy, Snapping scapula, Scapulothoracic bursitis

## Abstract

**Objective:**

Extracorporeal shockwave therapy (ESWT) has been used successfully in treatment of musculoskeletal disorders. Our objective was to assess the effectiveness of low versus middle-energy ESWT on snapping scapula bursitis.

**Methods:**

Thirty-five patients, divided into two groups, group (L), received low-energy ESWT, group (M) received middle-energy ESWT. Groups were evaluated at 1,3,6 and 12 months using the Visual Analogue Scale (VAS), the Constant-Murley scoring (CMS) and the Roles and Maudsley criteria.

**Results:**

In groups (L) and (M), VAS average values after 1,3,6 months and one year were (43±5.17, 38±4.33, 28±4.18 and 19±3.39) and (37±4.85, 26±4.74, 21±4.45 and 7±3.42) respectively. At six and twelve months, statistical difference was detected, P (0.034, 0.026) respectively. After one year of completing the treatment, the average values of CMS were (83.5±6.44 and 91±5.33) respectively, P=0.046. Roles and Maudsley criteria demonstrated that, patients in group (L), 6 (35%) excellent, 5 (29%) good, 4 (24%) acceptable and 2 (12%) had poor results. Whereas, patients in group (M), 11 (61%) excellent, 3 (17%) good, 3 (17%) acceptable and 1 (5%) had poor results.

**Conclusion:**

Although low-energy ESWT showed good early-term results, but middle-energy ESWT protocol demonstrated better early-term, Mid-term, and late-term results.

## INTRODUCTION

Extracorporeal shockwave therapy (ESWT) application in the treatment of musculoskeletal disorders has been increased within the last few years.^1^,^2^ Successful results were obtained in acute and chronic inflammatory conditions including lateral epicondylitis, planter fasciitis and calcific tendinitis of the shoulder.^3^,^4^

Snapping scapula is a condition first described by Boinet in 1867.^5^ Milch et al.^6^, had defined two distinct forms of scapulothorcic crepitus related to snapping scapula, the osseous form which is related to an bony pathology of the superomedial angle of the scapula such as an osteochondroma and the soft tissue form which is associated to inflammation of the bursa present around the superomedial angle of the scapula.

**Fig.1:**
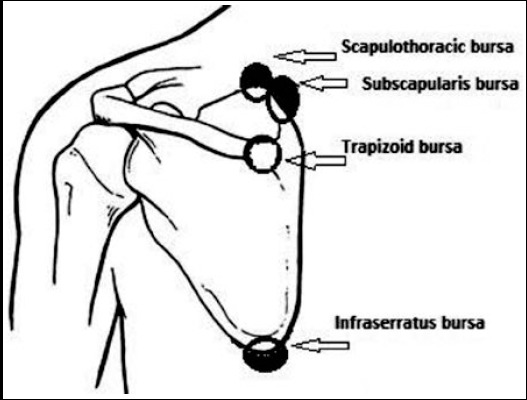
An anatomical diagram, showing the main periscapular bursae.

Kuhn et al described two types of bursal tissue scattered around the scapula, two major and four minor bursae. The major bursae are, the scapulothoracic bursa which is located between the posterior chest wall and the serratus anterior muscle and subscapular bursa which is located between the subscapularis and serratus anterior muscles (Fig.1). Over -use injuries of the muscles inserted around these two bursae or biomechanical dysfunction may lead to inflammation of these bursal tissue causing a painfull snapping scapular bursitis whereas the minor four bursae are scattered around the inferior margin of the scapula and the scapulothoracic articulation.^7^-^9^

**Fig.2:**
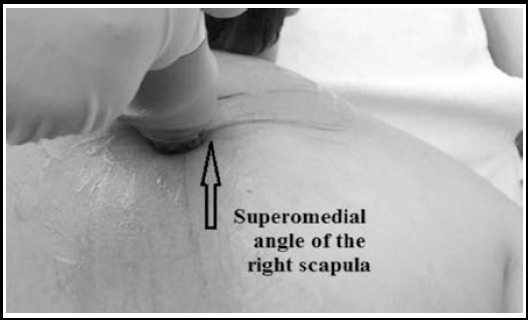
Application of extracorporeal shockwave therapy around the superomedial scapular angle while the patient in prone position.

Snapping scapula is commonly a misdiagnosed disorder.^5^ Most patients complain of pain at the superomedial angle of the scapula during activities, whereas others complain of pain even at rest.^7^ Usually the pain is the direct cause of the scapulothoracic bursitis located at the level of the levator scapulae muscle insertion at the superomedial angle of the scapula.^7^,^8^ Many treatment methods have been described to deal with snapping scapula, including surgical and non-surgical methods. Non-surgical measures consist of corticosteroid injection, modified extremity activity, rest with non-steroidal anti-inflammatory drug intake.^9^,^10^ ESWT has been described to give satisfactory results in comparison to corticosteroid injection in the treatment of painful cases of scapulothoracic bursitis.^11^

Different ESWT protocols have been described to treat variant musculoskeletal disorders including low, middle and high energy ESWT protocols.^1^-^4^,^11^ Middle energy ESWT protocol has been described previously to treat snapping scapula bursitis, demonstrated good early and late-term results. However, the applied middle energy ESWT protocol produced some complications like skin irritation, intramuscular hematomas and intermittent periscapular pain.^11^

The aim of this study was therefore to assess and compare the dose-related effect of two ESWT protocols, low-energy and middle-energy ESWT protocols on the treatment of snapping scapula bursitis.

## METHODS

Institutional ethical approval was granted by the ethical committee of research under the reference number 346-90. A prospective study was submitted on 35 patients diagnosed with scapulothoracic bursitis, divided into two groups according to the ESWT energy level applied. Group (L) composed of seventeen patients who received low-energy ESWT protocol and group (M) which is composed of eighteen patients who received middle-energy ESWT protocol. All demographic data related to patients involved in this study is demonstrated in detail in Table-I.

**Table-I:** Demographic data of patients involved in this study.

*ESWT groups*	*Group (L)*	*Group (M)*	*P value*
Gender			
Female (n)	12	15	
Male (n)	5	3	
Involved side			
Right (n)	9	13	
Left (n)	8	5	
Age average (y)	34.7± 6.4	36.2± 6.8	0.78

Patients with previously well-known cervical disc problems, congenital spine anomalies, neuromuscular disorders, rotator cuff pathologies, clotting disorder, infections and tumors were excluded from our study. The main purpose of this study was explained in detail for all patients before they accept and agree to be involved. To evaluate the pain associated with snapping scapula bursitis around the superomedial angle before and after completing the treatment protocol, patients were asked to quantify the pain severity level using the visual analogue scale method (VAS), which has been demonstrated to have a good construct validity and a valid measure of acute pain.^12^ Whereas, to evaluate the functional outcome of both treatment protocols reflection on the shoulder girdle, all patients were evaluated according to the Constant-Murley scoring (CMS) system. CMS combines physical tests (35 points) with subjective evaluation of the examined patient (65 points), which at the end of the evaluation, scores range between 0 (worst) and 100 (most favorable). CMS is the most commonly used method containing objective measurement and has been documented to be valid and reliable for many shoulder pathologies.^13^ However CMS is used in clinical practice to evaluate surgeries related to the glenohumeral joint related structures, specifically the rotator cuff surgeries.^14^,^15^ For this reason, the Roles and Maudsley criteria^16^, which measures the endpoint level of satisfaction for patients at the end of the follow up period, was added to the assessment methods. Although it has been used to assess the satisfaction level of the lower extremity surgery, however it was used before to estimate the satisfaction level of different treatment protocols.^11^,^16^

Roles and Maudsley criteria composed of four conditions depending mostly on measuring the residual pain compared to the pain before starting the treatment. Excellent; Patient has no pain with full movement and full activity, Good; Patient has occasional discomfort with full movement and full activity, Acceptable; Patient has some discomfort after prolonged activities, and Poor; Patient has a pain limiting activity.

All patients included in this study have a common complaint of posterior shoulder pain after overhead activities. Whereas thirteen patients described an audible crepitus accompanied with moderate to severe pain located at the superomedial scapular angle radiates mostly to the levator scapulae muscle. Twenty two patients did not describe audible crepitus. However physical examination demonstrated a crepitus during palpation and compression of the superomedial scapular angle against the posterior chest wall.

Radiological examinations, including plain radiographies, magnetic resonance imaging (MRI), computerized tomography (CT) and dynamic ultrasonographies were obtained to rule out any pathological condition that may mimic snapping scapula and to detect any fluid-filled small bursal lesions. However none of the radiological investigation was helpful in revealing inflamed bursal tissue.

To confirm our clinical diagnosis, which is based on severe pain on the superomedial scapular border squeezed to the posterior chest wall with a palpable crepitus and a pain with overhead activities radiating to the levator scapulae muscle, a 3 cc of local anesthetic (1% lidocain) was injected beneath the superomedial angle of the scapula. Partial or complete pain relief after the injection process was used as a strong indicator of the presence of an inflamed bursal tissue.^17^,^18^

The patients involved in this study were divided into two groups using the close envelope technique, group (L), the low-energy treatment protocol group, included seventeen patients, composed of 12 females and 5 males with an average age of 34.7± 6.4 years. Using the portable ESWT (BTL industries Ltd, UK) depending on the tolerance of patients, a weekly session for three weeks ranging from 5 to 7 minutes of (1500 pulses of 0.08 MJ/mm^2^), which is defined as a low-energy ESWT treatment protocol.^19^

Group (M), the middle-energy treatment protocol group, included eighteen patients, composed of 15 females and three males with an average age of 36.2± 6.8 years. A weekly session for three weeks ranging from 5 to 7 minutes of (2000 pulses of 0.2 MJ/mm^2^), which is defined as a middle-energy ESWT treatment protocol.^20^ The application of the ESWT was focused on the trigger and painful area around the superomedial angle of the scapula with the patients arm in extension, adduction and internal rotation with the patient in prone position (Fig.1).

All patients were asked to rest and reduce the level of the overhead activities as possible for one month during the ESWT application period, non-steroidal anti-inflammatory drugs were not allowed throughout the study period. All patients were followed clinically for at least one year. All patients were assessed clinically at one month, three months, six months and one year. After the end of treatment, the visual analogue scale (VAS) scores were recorded at each follow up. Whereas the level of satisfaction and functional outcome was evaluated for all patients using the Constant-Murley scoring (CMS) system and Roles and Maudsley criteria at the end of follow up. For statistical analysis the Mann-Whitney-U Test was used. The level of significance was set as p < 0.05.

## RESULTS

A total of 35 patients included in this study diagnosed with scapulothoracic bursitis (snapping scapula), divided into two groups, (L) group received low-energy ESWT protocol and (M) group received middle-energy ESWT protocol. All patients included in this study were evaluated one month, three months, six months and one year after completing the treatment protocol. The age averages for group (L) and group (M) were 34.7± 6.4 and 36.2± 6.8 years respectively. There was no statistical significance between the two groups regarding age with P= 0.78. Before starting the treatment the average VAS values were 78 ± 5.61 and 81 ± 5.92 respectively. No statistical significance was detected between the two groups with P value = 0.63. In group (L), after three sessions of weekly application of the low-energy ESWT protocol, the VAS average values after 1,3,6 months and one year were (43 ± 5.17, 38 ± 4.33, 28 ± 4.18 and 19 ± 3.39) respectively. Whereas in group (M), after three sessions of weekly application of the middle-energy ESWT protocol, the VAS average values after 1,3,6 months and one year were (37 ± 4.85, 26 ± 4.74, 21 ± 4.45 and 7 ± 3.42) respectively.

Although there was no significant difference between the study arms regarding the VAS average results after one and three months with P values (0.89 and 0.25) respectively. Group (M), which received middle-energy ESWT protocol, demonstrated low VAS average values compared to group (L) which received low-energy ESWT protocol. Six and twelve months after completing the treatment, both groups revealed a significant statistical difference in favor of group (M), regarding the VAS with P values (0.034, 0.026) respectively.

Before starting the treatment, the average values of the Constant-Murley scoring (CMS) system of group (L) and group (M) were (71.8 ± 9.36 and 73.4± 8.12) respectively. No statistical significance was detected between the two study arms with P= 0.18. However, one year after completing the treatment, the average values of the Constant-Murley scoring system were (83.5 ± 6.44 and 91± 5.33) respectively. A statistical significance was detected between the two groups with P= 0.046. Roles and Maudsley criteria, which was used mainly to assess the satisfaction level of the tow treatment protocols demonstrated that, out of 17 patients in group (L), six patients (35%) had excellent, 5 patients (29%) had good, 4 patients (24%) had acceptable and two patients (12%) had poor results. Whereas, out of 18 patients in group (M), 11 patients (61%) had excellent, three patients (17%) had good, 3 patients (17%) had acceptable and one patient (5%) had poor results (Table-II).

**Table-II:** Subjective and objective evaluation of the two treatment protocols. The clinical out-come produced by the study groups in patients treated with different ESWT protocols

*Clinical out-come variables*	*Group (L)*	*Group (M)*	*P value*
Age average before treatment	78 ± 5.61	81 ± 5.92	0.63
VAS (1 month)	43 ± 5.17	37 ± 4.85	0.89
VAS (3 months)	38 ± 4.33	26 ± 4.74	0.25
VAS (6 months)	28 ± 4.18	21 ± 4.45	0.034
VAS (12 months)	19 ± 3.39	7 ± 3.42	0.026
Constant-Murley scoring average values			
Before treatment	71.8 ± 9.36	73.4± 8.12	0.18
After one year	83.5 ± 6.44	91± 5.33	0.046
Roles Maudsley criteria	Out of 17 patients	Out of 18 patients	
Excellent	6 patients (35%)	11 patients (61%)	
Good	5 patients (29%)	3 patients (17%)	
Acceptable	4 patients (24%)	3 patients (17%)	
Poor	2 patients (12%)	1 patient (5%)	

ESWT:energy extracorporeal shockwave therapy, VAS:visual analogue scale.

## DISCUSSION

Many treatment methods have been described for the treatment of a resistant snapping scapula ranging from surgical excision of the superomedial border of the scapula to exercise and activity modification. However, non-invasive methods have been described to be ineffective in dealing with the pain and crepitus of the scapulothoracic bursitis.^21^

The precise mechanisms of ESWT action is still unknown. Loew et al.^22^, described three modes of action of ESWT, mechanical effect that results in deposit fragmentation, molecular effect that results in deposit phagocytosis and analgesic effect results in denervation of pain receptors. ESWT can be divided into three categories based on the level of energy produced. Low–energy (< 0.08 mJ/mm^2^), middle-energy (0.08–0.28 mJ/mm^2^), and high-energy (> 0.28 mJ/mm^2^).^23^

The use of ESWT in treatment of soft tissue injuries and tendon lesions has been highly controversial, it has been described to be very beneficial in many musculoskeletal system disorders.^24^ Rompe et al.^25^ reported that, in general, the ESWT trials conducted on soft tissue disorders such as planter fasciitis, lateral epicondylitis and calcific tendenitis of the shoulder most of the time use a 1500 – 3000 shocks of a low-energy protocol applied mostly on the site of maximum tenderness and it is usually applied three to five times, once every week.

ESWT was found to induce a long term tissue regeneration effect and produces an immediate analgesic and anti-inflammatory outcomes. Chemical inflammation mediators washout and triggering of neovascularization together with nociceptive inhibition are described to be the essential biological effect of ESWT on tissue.^26^,^27^

In in-vitro studies, it had been demonstrated that ESWT at low and middle energy field density (EFD) is documented to produce a neoangiogenic effect by increasing the expression of vascular endothelial growth factor and its receptor Flt-1.^28^ Gotte et al demonstrated that ESWT induces a nonenzymetic production of nitric oxide and a subsequent suppression of NF- B (nuclear factor kappa B) activation which are responsible for the clinically beneficial effect of ESWT on tissue inflammation.^28^,^29^

Several studies have demonstrated that high energy ESWT protocols applied to calcific tendenitis of the rotator calf led to improvement of the muscle function and shrinkage of the subacromial bursa and thus improved the clinical picture of the involved shoulder. 30,31 There are high-energy, middle-energy, and low-energy ESWT protocols used in clinical applications of musculoskeletal disorders, However to date, it is still not clear which level of energy is more effective in pain relief and clinical improvement of pain caused by inflamed bursa and tissue degeneration.^25^,^32^

Searching the literature revealed only a single study that used a middle-energy ESWT protocol to deal with snapping scapula. ESWT has been demonstrated to be superior to corticosteroid injection in treating snapping scapula related scapulothoracic bursitis.^11^ However in that study, ESWT of 0.1 to 0.15 MJ/mm^2^ energy protocol with 1500 to 2500 pulses of 1.4 to 2.1 bars, were used. Which is a middle-energy protocol.^24^,^25^

The middle-energy and low-energy ESWT protocols have been used frequently in musculoskeletal disorders. Middle- energy ESWT protocol is commonly used in calcific tendinitis of the shoulder joint, trochanteric bursitis and calcaneal bursitis.^16^,^24^,^25^ Whereas the low-energy ESWT protocol is frequently used in lateral epicondilitis, pes anserine bursitis and planter fasciitis.^17^,^18^ This study shows that, although there was no statistical difference between the low-energy and middle-energy ESWT protocols at early-term (one month) and mid-term (three months) results, however late-term results demonstrated statistical significance at six and twelve months in favor of the middle-energy ESWT.

The functional results evaluated by the Constant-Murley scoring system, did not show any statistical significance between the two groups prior to treatment induction. However, after one year of completing the treatment protocol, group (M) demonstrated higher and statistically significant score average than that of group (L). However, Constant-Murley scoring system, despite its being the most commonly used scoring system for shoulder functional evaluation recommended by the European Society of Elbow Surgery,^18^,^19^ it is designed more specifically to evaluate lesions of the rotator cuff muscles and surgeries related to them. For that reason the average scores of the pretreatment period were high, since the rotator cuff pathology was one of the exclusion criteria of this work, thus all patients involved in this study were rotator cuff pathology free.

To precisely evaluate the treatment satisfaction level after one year, the Roles Maudsley criteria was used. In group (M), 78% of patients showed excellent to good results, whereas 64% of patients in group (L) demonstrated excellent to good results.

Although both ESWT protocols showed to be effective in treating snapping scapula associated with scapulothoracic bursitis, however middle-energy ESWT demonstrated superior results.

During the early treatment period, just few skin irritation and minimal skin burning sensation were noticed in the low-energy protocol which resolved within a couple of days. However, in the middle-energy protocol, besides skin irritation and burning sensation recognized in some patients a localized minimal muscle hematomas were detected in three patients, resolved within a week.

High-energy ESWT protocol in which the energy exceeds 0.28 mJ/mm^2^ has been used by many researchers to treat calcific tendinitis of the rotator calf muscles and avascular necrosis of the femoral head.^24^,^25^ However, high-energy ESWT is more painful and requires intravenous analgesia. In addition to that, due to the high energy applied, few complications like humerous avascular necrosis and large muscular hematomas.^22^-^25^ Although, ESWT has been applied for ischemic heart disease with low-energy protocols^33^, we found it hazardous to apply this protocol to the superomedial angle of the scapula due to its adjacent relationship to many vital structures like the pleura of the lung and the large chest vasculature.

### Limitations of the study

The number of the involved patients is small. Since snapping scapula bursitis is an underestimated condition, yet there is no specific subjective and objective criteria that can evaluate the scapular movement function apart.

## CONCLUSION

Low and middle-energy ESWT protocols can be safely and successfully used for the treatment of snapping scapula associated with scapulothoracic bursitis. Although low-energy ESWT showed good early-term results, but middle-energy ESWT protocol demonstrated better early-term, Mid-term, and late-term results.
